# P51 The patient perspective on IV and oral antibiotics

**DOI:** 10.1093/jacamr/dlaf118.058

**Published:** 2025-07-14

**Authors:** Amirah Hussain, Isobel Robinson

**Affiliations:** Northern Care Alliance, UK; Northern Care Alliance, UK

## Abstract

**Objectives:**

To explore the patient perspective on the administration of IV and oral antimicrobials.

**Background:**

Improving IV to Oral antimicrobial switch (IVOS) rates facilitates patient flow, reduces nursing time spent reconstituting iv antibiotics, increases the use of cost-effective antibiotics, reduces the ward level carbon footprint, and decreases the risk of developing line related infections. This work aims to explore patient experience, perception and views, in a secondary care setting, around the administration routes of antimicrobials.

**Methods:**

After patient consent, a semi-structured interview comprising both open and closed ended questions, was conducted. This allowed clarification or further explanation of answers, providing mid-level insights. Interviews were carried out with 31 patients who were randomly selected across medical wards over one week. Eligible participants were required to have mental capacity, must have received both IV and oral antibiotics for at least 48 h during their hospital admission, and consented to participate in the interview.

**Results:**

Oral antibiotics were preferred by 67% (16/24) of patients, as they enabled them to ‘move around and go to the shops’, they can ‘go home on them’, and many expressed a preference for avoiding needles when possible, stating they would ‘rather swallow a tablet than have a needle’. Those who preferred IV antibiotics felt IVs helped them ‘get better quicker’, and a patient felt ‘oral antibiotics disturbed my stomach’. When asked whether patients felt IV antibiotics were more effective than oral, 32% (12/31) patients were unsure, as ‘they did not know how they work’, 23% (7/31) patients felt that IV were not more effective as ‘they are the same thing’ and ‘oral is better’, and 39% (12/31) patients felt that IV antibiotics were more effective as they ‘go straight into the bloodstream’, and ‘have worked in the past’. When asked if they required more information on their prescribed antibiotics, 8/31 (23%) of patients felt they had been adequately informed. From the sample of 31 patients, 74% of participants expressed how they would like more information around the antibiotics prescribed for them, why and how they are given to them while in hospital.

**Conclusions:**

Providing patients with the opportunity to discuss and receive additional information regarding antimicrobials prescribed would be valued. The patients interviewed have built perceptions around antibiotic administration routes based on previous experiences. Patient targeted approaches to antimicrobial stewardship would be well received, potentially challenging existing beliefs and empowering patients.

